# Computational analysis of cas proteins unlocks new potential in HIV-1 targeted gene therapy

**DOI:** 10.3389/fgeed.2023.1248982

**Published:** 2024-01-04

**Authors:** Will Dampier, Rachel Berman, Michael R. Nonnemacher, Brian Wigdahl

**Affiliations:** ^1^ Department of Microbiology and Immunology, Drexel University College of Medicine, Philadelphia, PA, United States; ^2^ Center for Molecular Virology and Gene Therapy, Institute for Molecular Medicine and Infectious Disease, Drexel University College of Medicine, Philadelphia, PA, United States; ^3^ Sidney Kimmel Cancer Center, Thomas Jefferson University, Philadelphia, PA, United States

**Keywords:** gene therapy, CRiSPR/Cas, HIV-1, guideRNA, cure strategy

## Abstract

**Introduction:** The human immunodeficiency virus type 1 (HIV-1) pandemic has been slowed with the advent of anti-retroviral therapy (ART). However, ART is not a cure and as such has pushed the disease into a chronic infection. One potential cure strategy that has shown promise is the Clustered Regularly Interspaced Short Palindromic Repeats (CRISPR)/Cas gene editing system. It has recently been shown to successfully edit and/or excise the integrated provirus from infected cells and inhibit HIV-1 *in vitro*, *ex vivo*, and *in vivo*. These studies have primarily been conducted with SpCas9 or SaCas9. However, additional Cas proteins are discovered regularly and modifications to these known proteins are being engineered. The alternative Cas molecules have different requirements for protospacer adjacent motifs (PAMs) which impact the possible targetable regions of HIV-1. Other modifications to the Cas protein or gRNA handle impact the tolerance for mismatches between gRNA and the target. While reducing off-target risk, this impacts the ability to fully account for HIV-1 genetic variability.

**Methods:** This manuscript strives to examine these parameter choices using a computational approach for surveying the suitability of a Cas editor for HIV-1 gene editing. The Nominate, Diversify, Narrow, Filter (NDNF) pipeline measures the safety, broadness, and effectiveness of a pool of potential gRNAs for any PAM. This technique was used to evaluate 46 different potential Cas editors for their HIV therapeutic potential.

**Results:** Our examination revealed that broader PAMs that improve the targeting potential of editors like SaCas9 and LbCas12a have larger pools of useful gRNAs, while broader PAMs reduced the pool of useful SpCas9 gRNAs yet increased the breadth of targetable locations. Investigation of the mismatch tolerance of Cas editors indicates a 2-missmatch tolerance is an ideal balance between on-target sensitivity and off-target specificity. Of all of the Cas editors examined, SpCas-NG and SPRY-Cas9 had the highest number of overall safe, broad, and effective gRNAs against HIV.

**Discussion:** Currently, larger proteins and wider PAMs lead to better targeting capacity. This implies that research should either be targeted towards delivering longer payloads or towards increasing the breadth of currently available small Cas editors. With the discovery and adoption of additional Cas editors, it is important for researchers in the HIV-1 gene editing field to explore the wider world of Cas editors.

## 1 Introduction

The CRISPR/Cas system has revolutionized the field of genetic engineering and biotechnology with applications to provide a cure to genetic diseases affected at a single locus as well as people living with human immunodeficiency virus type 1 (HIV-1) (Liao et al., 2015; Atkins et al., 2021). The CRISPR (Clustered Regularly Interspaced Short Palindromic Repeats) system is a naturally occurring mechanism found in bacteria and archaea which serves as an adaptive immune system, enabling these organisms to defend against viral infections by storing and recognizing fragments of viral DNA. The Cas (CRISPR-associated) protein is a key component of the CRISPR system which acts as an effector molecule that carries out the DNA editing/cleaving function and is accompanied by the guide RNA (gRNA) which acts as a targeting scaffold for the Cas protein to interact with and recognize DNA and RNA targets via complementary base pairing.

Cas proteins are divided into two main classes: Class 1 systems use multiple Cas proteins while Class 2 effectors use a single, multidomain protein with all activities required for DNA/RNA interference. This makes them ideal for use in biomolecular delivery and genetic engineering. The Cas protein classes are further subdivided into types based on distinctive signatures and architecture of the Cas endonucleases; Class 1 systems include types I, III, and IV, while Class 2 systems contain types II (Cas9), V (Cas12), and VI (Cas13). Type II Cas9 effectors contain RuvC and HNH nuclease domains while type V Cas12 proteins have a single RuvC domain and type VI Cas13 proteins lack a DNase domain but have two conserved HEPN domains involved in RNA cleavage (Shmakov et al., 2017; Konermann et al., 2018; Zhang et al., 2019a; Yan et al., 2019; Makarova et al., 2020).

Cas proteins surveil the genome randomly and recognize a specific sequence of nucleotides referred to as the protospacer adjacent motif (PAM) site. Upon PAM recognition, base-pairing between the adjacent target site and the 20 bp protospacer sequence of the gRNA provides the programable targeting to a specific instance of a PAM site on the genome (Doench et al., 2014). Upon sufficient gRNA:target homology, the Cas protein cuts genomic DNA in either a blunt (type II effectors) or staggered (type V effectors) fashion. Engineering this system to target a specific genomic locus involves finding a suitable PAM site within the target of interest and constructing a gRNA to match the adjacent region. The PAM site of an effector is an important design consideration when targeting a genomic locus as it limits the space of targetable locations. Recent advances in molecular engineering of the Cas effector have targeted expanding the available PAM sites.

Gene length is another important consideration when choosing a Cas effector. The type II Cas9 from *Streptococcus pyogenes* (SpCas9) is the most well characterized Cas system and has been effectively used in gene therapy, but in terms of biomolecular delivery the SpCas9 is 4,104 bases in length which limits compact delivery systems like adeno-associated viral (AAV) vectors with a carrying capacity of 4.7 kilobases (Cong et al., 2013; Hsu et al., 2013). This has fueled research in the Cas9 ortholog from *Staphylococcus aureus* (SaCas9) which is markedly smaller than SpCas9 at 3,159 bases (Nishimasu et al., 2015; Ran et al., 2015). However, SaCas9 recognizes a restrictive PAM site of NNGRRT limiting its targeting potential relative to the shorter NGG of SpCas9. The type V Cas12 effectors, previously known as Cpf1, recognize T-rich PAM sites. Several of these from *Lachnospiraceae bacterium* (LbCas12a), *Acidaminococcus sp.* (AsCas12a), and *Francisella novicida* (FnCas12a) have shown potential in human cells (Zetsche et al., 2015; Hirano et al., 2016a; Dong et al., 2016; Yamano et al., 2016; Acharya et al., 2019). The type VI Cas13 systems have almost no PAM requirement and target RNA for degradation (Abudayyeh et al., 2017).

In attempts to generate both compact and broad effector Cas proteins, scientists have deepened the search into Cas orthologs in a bid to identify Cas effectors that are small and have PAM sites that allow for more flexibility (Esvelt et al., 2013; Shmakov et al., 2015; Burstein et al., 2017; Shmakov et al., 2017; Harrington et al., 2018; Gasiunas et al., 2020; Hu et al., 2020; Wang et al., 2020). To this end, variants of the Cas proteins have been engineered through base-editing the amino acids around the PAM recognition domain to enhance the efficiency of the Cas protein, such as HypaCas9 and eSpCas9 (Slaymaker et al., 2016; Tycko et al., 2016; Schmid-Burgk et al., 2020), or to alter the PAM recognition site of the Cas effector, such as SpCas9-NG or SaCas9-KKH which recognize NG and NNNRRT, respectively (Kleinstiver et al., 2015a; Kleinstiver et al., 2015b; Anders et al., 2016; Hirano et al., 2016b; Gao et al., 2017; Hu et al., 2018; Yuen et al., 2022; Spasskaya et al., 2023). Therefore, the Cas system has significantly expanded as a toolbox for targeting DNA or RNA and these orthologs and variants of Cas effectors have yet to be fully explored for their use in targeting the HIV-1 provirus.

Both HIV-1 viral and host components, such as the co-receptors CCR5 and CXCR4 (Allen et al., 2018), have been targeted in HIV-1 cure strategies, but the integrated proviral DNA remains as the main target to eliminate the latent viral reservoirs (Liu et al., 2017c; Yin et al., 2017; Xiao et al., 2019b). The high variability of HIV-1 sequences both within and among patients requires careful engineering of gRNAs which are both safe and effective as well as broad-spectrum (Sullivan et al., 2019; Chung et al., 2020; Sullivan et al., 2020; Allen et al., 2023). HIV-1-specific gRNAs have been designed which are capable of broadly targeting quasispecies, and now we further our exploration into CRISPR/Cas system engineering by querying potential orthologous and/or variant Cas proteins for targeting HIV-1. The earliest methodologies considered the sequence of a single lab-strain (Hu et al., 2014; Dampier et al., 2017). These evolved to consider genetic variability across databases (Roychoudhury et al., 2018), within single individuals (Dampier et al., 2018), and across subtypes (Chung et al., 2021).

Computational selection of gRNAs is a burgeoning field with tools available for most purposes. End-to-End tools like Benchling (2023) and CHOPCHOP (Labun et al., 2019) are able to guide a researcher through the selection and *in silico* screening of high-quality gRNAs. However, they are limited to pre-defined Cas and genome searches and cannot be tuned to nominate from one genome and evaluate off-targets in a different genome. Tools like Crisflash (Jacquin et al., 2019) can be used to find targets in variable sequences through the use of a Variant Call Format (vcf) file; however, this tool is tuned to mammalian genetics and cannot handle a quasispecies as a list of phases. Other tools like CRISPR MultiTargeter (Prykhozhij et al., 2015) are designed to find a protospacer that is repeated multiple times across a transcript to improve targeting. None of these, have the required features for the proposed analysis. This manuscript introduces a new paradigm in searching for Cas targets in HIV-1 to address these issues. We call this pipeline the: Nominate, Diversify, Narrow, Filter (NDNF) pipeline.

## 2 Results

### 2.1 Cas editor literature review

The literature search was conducted by reviewing publications from PubMed and Google Scholar with the queries for CRISPR and Cas as well as current and previous nomenclature for individual class 2 Cas proteins, HIV-1 and CCR5/CXCR4 (last searched 6/23/2023) and all relevant articles were reviewed. Information relevant to gene editing potential was collected into Table 1 and Supplementary Table S1. This includes information like the name, source organism, PAM recognition site, protospacer size, gene size, induced mutations, and specific references related to current application in HIV-1.

**TABLE 1:** Cas effector protein variants and orthologs with potential for use in targeting HIV-1.

Type	Effector/Name	Size (aa)	Spacer (nt)	PAM/PFS	Target	DSB	HIV-1 target	Host target	Ref.
II-A	SpCas9	1,368	20	5'-(PS)-NGG	dsDNA	Blunt	Ebina et al. (2013), Hu et al. (2014), Liao et al. (2015), Zhu et al. (2015), Wang et al. (2016a), Kaminski et al. (2016b), Wang et al. (2016b), Kaminski et al. (2016c), Wang et al. (2016c), Yin et al. (2016), Lebbink et al. (2017), Ophinni et al. (2018), Chung et al. (2020), Ophinni et al. (2020), Sessions et al. (2020), Herskovitz et al. (2021), Khanal et al. (2022), Allen et al. (2023)	CCR5/CXCR4 (Li et al., 2015; Liu et al., 2017c; Ehrke-Schulz et al., 2017; Xu et al., 2017; Knipping et al., 2022; Li et al., 2022; Scheller et al., 2022)	Cong et al. (2013), Hsu et al. (2013), Mali et al. (2013), Ran et al. (2013), Nishimasu et al. (2014), Wu et al. (2014)
								APOBEC3G/APOBEC3B (Bogerd et al., 2015)	
								Tetherin (Zhang et al., 2019b)	
II-A	SpCas9-NG	1,368	20	5’-(PS)-NG	dsDNA	Blunt	-	CCR5/CXCR4 (Knipping et al., 2022)	Nishimasu et al. (2018), Kim et al. (2020), Oura et al. (2021), Sangree et al. (2022)
II-A	SaCas9	1,053	21–23	5'-(PS)-NNGRRT	dsDNA	Blunt	Kaminski et al. (2016a), Yin et al. (2017), Wang et al. (2018), Dash et al. (2019)	CCR5/CXCR4 (Wang et al., 2017; Xiao et al., 2019a; Dash et al., 2023)	Nishimasu et al. (2015), Ran et al. (2015)
II-A	SaCas9-KKH	1,053	20–23	5'-(PS)-NNNRRT	dsDNA	Blunt	-	CCR5 (Liu et al., 2021a)	Kleinstiver et al. (2015a), Yuen et al. (2022)
V-A	LbCas12a (Cpf1)	1,228	23–25	5′-TTTV-(PS)	dsDNA; ssDNA	5′ staggered	Gao et al. (2020), Fan et al. (2022), Krysler et al. (2022)	-	Dong et al. (2016), Yamano et al. (2017), Chen et al. (2018)
V-A	AsCas12a (Cpf1)	1,307	24	5′-TTTN-(PS)	dsDNA; ssDNA	5′ staggered	Liu et al. (2021b)	CCR5 (Liu et al., 2020)	Yamano et al. (2016), Yamano et al. (2017), Chen et al. (2018)
V-B	AaCas12b (C2c1)	1,129	20	5′-TTN-(PS)	dsDNA	5′ staggered	-	CCR5 (Teng et al., 2018)	Shmakov et al. (2015), Teng et al. (2018)
V-E	Cas12e (PlmCasX2)	978	16–24	5′-TTCN-(PS)	dsDNA	5′ staggered	-	CCR5 (Armstrong et al., 2023)	Burstein et al. (2017), Liu et al. (2019), Yang and Patel (2019), Selkova et al. (2020)
VI-A	LbuCas13a (C2c2)	1,159	24	No PFS requirement	ssRNA	N/A	Yin et al. (2020)	-	Liu et al. (2017b)
VI-D	RspCas13d (CasRx)	922	20	No PFS requirement	ssRNA	N/A	Nguyen et al. (2021)	-	Konermann et al. (2018), Yan et al. (2018), Zhang et al. (2018), Zhang et al. (2019a), Cheng et al. (2023)

N/A, not applicable; nt, nucleotide(s); aa, amino acids; PAM, protospacer adjacent motif; PFS, protospacer flanking sequence PS, protospacer; N, any base; R = A/G; Y = C/T; W = A/T;V = A/C/G; H = A/C/T; D = A/G/T; M = A/C; B = G/T/C.

Using this literature review as a guide, a computational pipeline was developed to interrogate each enzyme on the targetable space of HIV-1. This pipeline simplifies each enzyme to a PAM recognition site search. While this removes knowledge about position specific mismatch tolerance, that information is unknown for novel enzymes. The intent is instead, to consider each PAM equally and understand how that engineering choice impacts the targetable space of HIV-1.

### 2.2 Cas editor choice influences the targetable HIV-1 genome

In brief, the NDNF pipeline considered all possible gRNAs nominated from a diverse set of HIV-1 sequences. These were further diversified through random mutations to a target pool size of 40,000. The pool was narrowed by comparison to an independent, globally representative set of HIV-1 genomes, labeling those capable of targeting >=75% of the sequences as *broad*. Finally, the gRNAs were filtered through a mismatch search against the human genome labeling only those with no hits as *safe*. Finally, in order to estimate the impact of mutations at a site, we considered an HIV-1 mutation dataset that interrogated the impact of point mutations on replication capacity to label gRNAs in areas of lethal mutations as *effective*. From this pipeline, each of the 40,000 potential gRNAs for each PAM was labeled as broad, safe, or effective, neither, or any combination. For the purposes of ranking and describing enzymes, we considered the number of gRNAs that were safe and broad and effective and termed them SBE gRNAs. The details of this pipeline are discussed in the Methods section.

**FIGURE 1:**
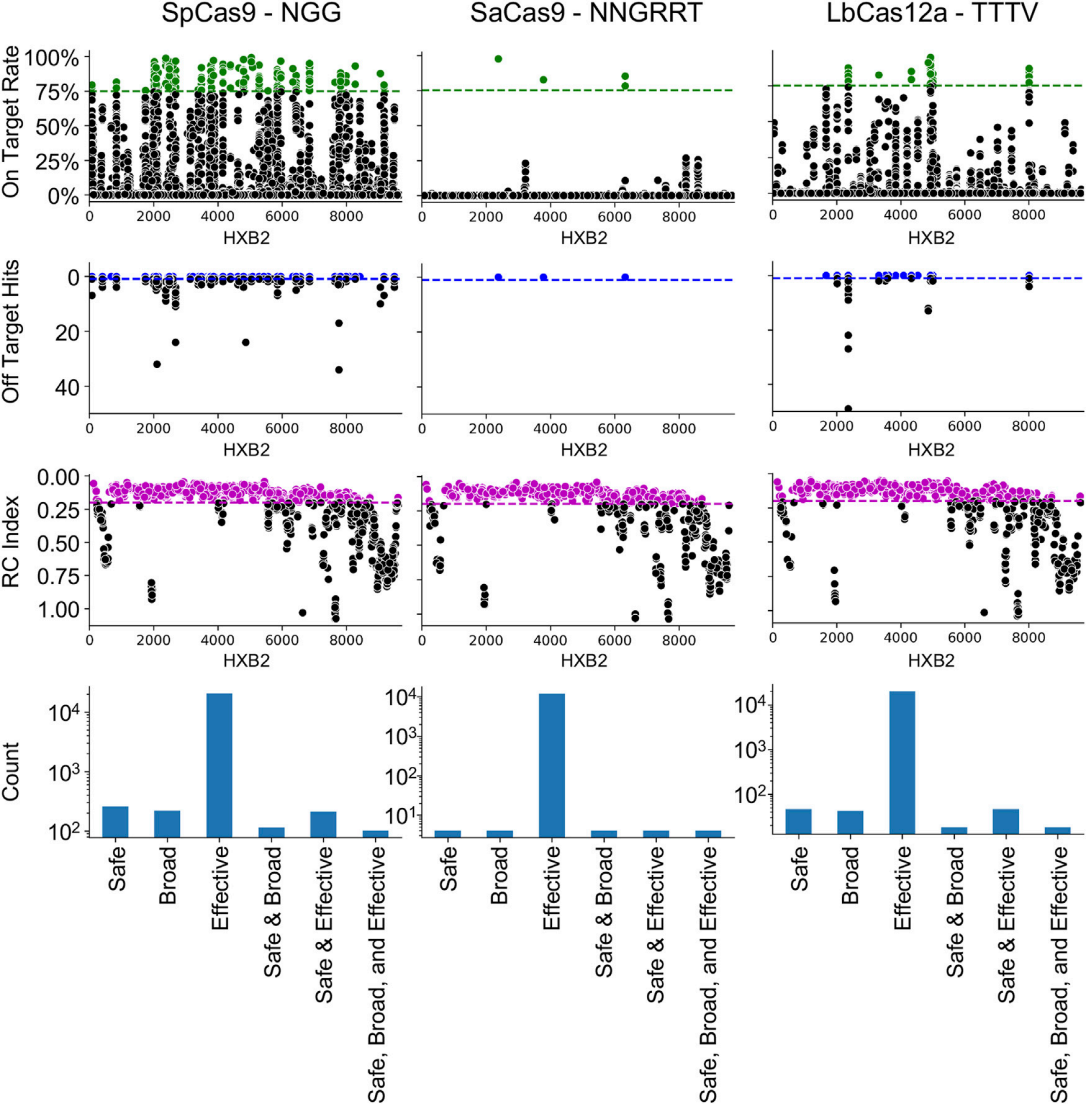
Cas9 editor influences the number of safe, broad, and effective (SBE) targets. Each column indicates the results of the NDNF pipeline for SpCas9 (left), SaCas9 (middle), and LbCas12a (right). The PAM sequence used is indicated in the graph labels. The top row indicates the broad gRNAs by plotting the on-target rate for each gRNA, those in green are above a 75% cutoff and considered broad. The second row indicates the safety of each gRNA by plotting the off-target count for each gRNA. Those with no hits are considered safe (above the blue line). The third row indicates the effectiveness of each gRNA by plotting the average RC index of the 40 bp window centered on the cut-site. The fourth row plots the counts of SBE gRNAs and their overlaps.

The NDNF pipeline was first used on the three most common editors used in HIV-1 gene editing: SpCas9, SaCas9, and LbCas12a (Figure 1). The results demonstrated that the locations on HXB2 that are broadly targetable by the three different enzymes varies with SpCas9 having the largest number of SBE gRNAs (SBE = 75). Conversely, SaCas9, while having fewer broad targets, presented a better safety profile with fewer off-target risks (SBE = 9). LbCas12a had only 10 SBE gRNAs under this metric. All three enzymes target regions of lethal mutations across the HIV-1 genome (Figure 1: RC index).

### 2.3 Mismatch tolerance is a double-edged sword

**FIGURE 2:**
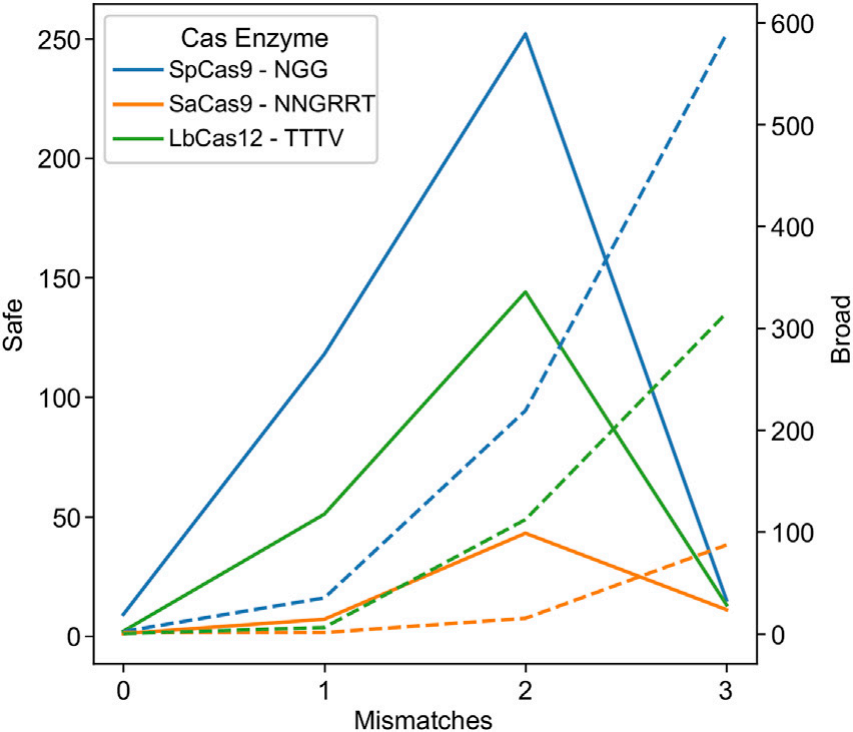
Higher promiscuity leads to more broad targets but fewer safe ones. The number of safe gRNAs was calculated and plotted for the wild-type PAMs indicated in the key for SpCas9 (blue), SaCas9 (orange), and LbCas12a (green) in solid lines using the left axis. The number of broad gRNAs was calculated and plotted for the wild-type PAMs for SpCas9 (blue), SaCas9 (orange), and LbCas12a (green) in dotted lines using the right axis.

Researchers looking to reduce the risk of off-target effects have engineered mutations in the enzyme (Kleinstiver et al., 2016; Zheng et al., 2018; Kim et al., 2023) or the sgRNA handle (Gupta et al., 2021) to require a tighter coupling between gRNA:target pairs before cleavage. This reduces the likelihood of an off-target effect, but conversely reduces the tolerance to HIV-1 genetic variability. In order to investigate this phenomenon and its impact on HIV-1 targeting, an experiment was performed where the mismatch tolerance was varied between 0 and 3 (Figure 2).

As hypothesized, low mismatch values led to few broad gRNAs, due to a decrease in the number of gRNAs that could target 75% of the validation dataset at a strict mismatch tolerance (Figure 2: dotted lines). Conversely, mismatch tolerances of three or greater led to a decrease in safe gRNAs, due to an ability to target increasingly distantly homologous human sites (Figure 2: solid lines). The ideal tradeoff between safe and broad was determined to be two mismatches for SpCas9, SaCas9, and LbCas12a, as shown by the peak in the solid lines in Figure 2.

### 2.4 Modifications to PAM tolerance improves targetable sites

**FIGURE 3:**
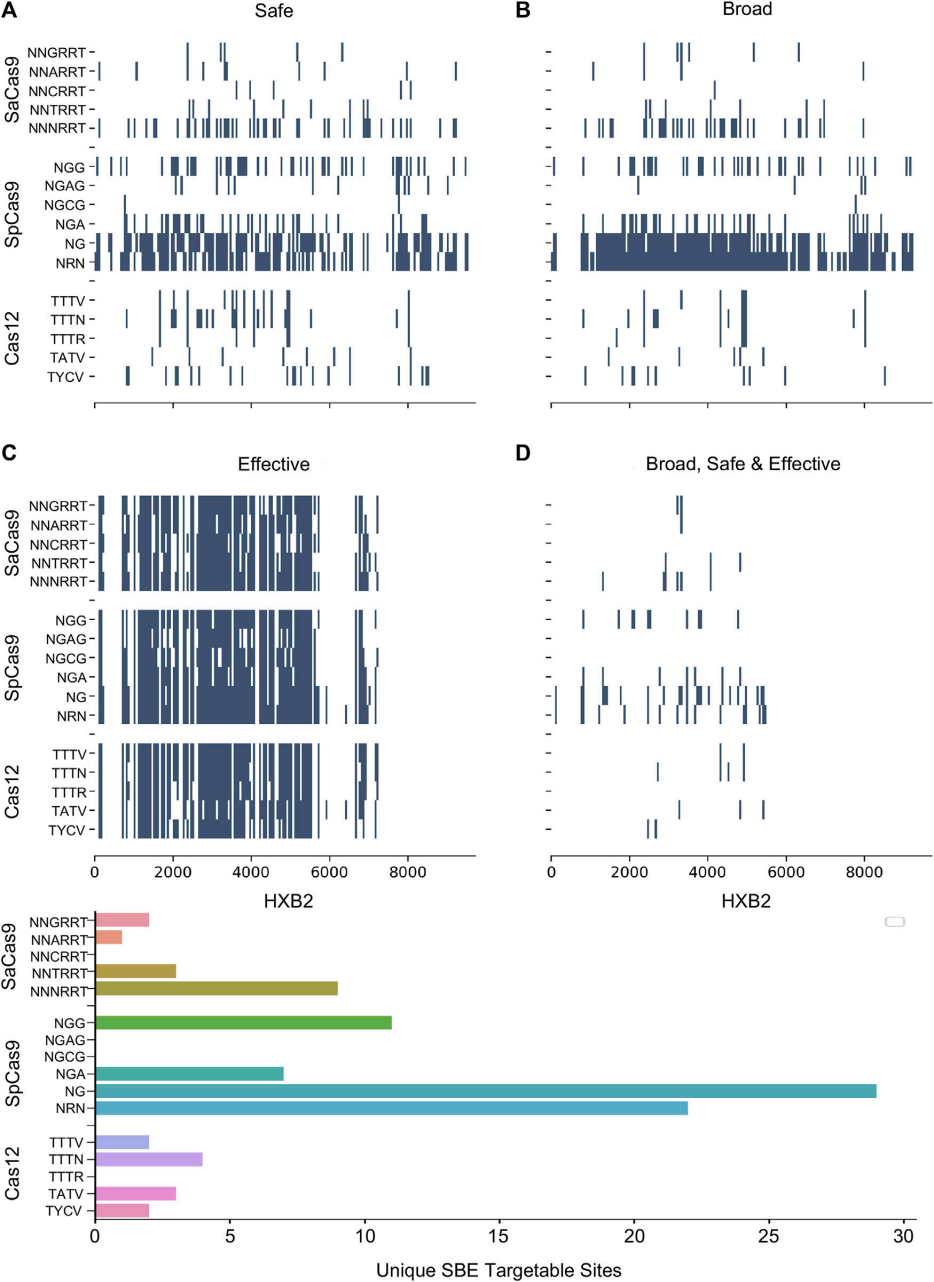
The pattern of targetable regions is altered by PAM mutations. For each of the SpCas9, SaCas9, and LbCas12a variants identified, the pattern of broad **(A)**, safe **(B)**, and effective **(C)** gRNA is plotted against the target position in the HXB2 reference genome. **(D)** Indicates the overlapping sites of safe, broad, and effective gRNAs. The counts of each category are provided in Table 2. E) A bar graph showing the number of unique genomic positions targetable by SBE gRNAs for each PAM choice.

Numerous studies have generated Cas editor variants with altered PAM recognition sites described in Table 1. This can either be to broaden or restrict sites to increase targetable space or reduce off-target effects, respectively (Kleinstiver et al., 2015b; Hu et al., 2018; Zhang et al., 2021; Ma et al., 2022). The known modified PAMs for SpCas9, SaCas9, and LbCas12a were interrogated using the same NDNF pipeline (Figures 3A–D; Table 2). In each case, the broadening of the PAMs led to an increase in the number of targetable sites across the HIV-1 genome (Figure 3E). SaCas9 gains 28 new SBEs (9–37, Table 2) and can target 7 additional sites (Figure 3E) by moving from the NNGRRT to NNNRRT. LbCas12a gains 9 SBEs and 2 additional sites moving from TTTV to TTTN. SpCas9 has a mixed effect by moving to broader editors; changing from NGG to NG decreases the number of SBEs 75 *versus* 41 but increases the number of targetable sites from 11 to 29. Adjusting to the even broader NRN motif loses more SBEs (75 vs. 24) and only increases number of sites to 22.

**TABLE 2:** The number of safe, broad, and effective (SBE) gRNAs found by the NDNF pipeline.

Cas	PAM motif	Safe	Broad	Effective	SBE	Unique sites
ASp2Cas12l	CCY	17	255	6,696	1	1
AacCas12b	TTN	6	1,047	8,946	1	1
AsCas12a	TTTN	14	329	8,427	3	2
Asp2Cas12l	CCY	11	267	6,742	2	1
BthCas12b	ATTN	12	135	7,379	8	2
Cas12c2	TN	1	5,207	9,606	1	1
Cas12d	TA	7	850	8,809	0	
Cas12e	TTCN	19	83	6,313	0	
Cas12h1	RTR	1	615	8,471	1	1
Cas12j	TBN	12	3,534	9,333	0	
CasY	TA	9	871	8,785	1	1
CjeCas9	NNNRYAC	7	11	5,695	0	
Cpf1-TATV	TATV	41	21	6,981	6	3
Cpf1-TTTN	TTTN	132	105	8,573	19	4
Cpf1-TTTR	TTTR	48	35	6,710	0	
Cpf1-TTTV	TTTV	55	43	7,464	10	2
Cpf1-TYCV	TYCV	142	50	6,337	2	2
ErCas12a	YTTN	17	603	8,310	1	1
FnCas12a	TTV	8	529	7,900	1	1
FnCas9-RHA	YG	8	810	8,150	0	
GeoCas9	NNNNCRAA	0	1	4,754	0	
LbCas12a_Cpf1	TTTN	212	1,051	41,970	26	4
Nme1Cas9	NNNNGATT	6	0	4,590	0	
OspCas12c	TG	11	595	7,912	0	
SaCas9-KKH-A	NNARRT	43	22	6,942	1	1
SaCas9-KKH-C	NNCRRT	33	2	6,043	0	
SaCas9-KKH-N	NNNRRT	236	227	8,808	37	9
SaCas9-KKH-T	NNTRRT	45	17	7,102	6	3
SaCas9-WT	NNGRRT	103	260	42,779	9	2
SauriCas9	NNGG	27	600	7,841	5	4
SauriCas9-KKH	NNRG	11	1,936	8,506	1	1
ScCas9	NNG	11	2,773	9,022	2	2
SpCas9-EQR	NGAG	62	22	5,033	0	
SpCas9-NG	NG	365	1,291	8,879	41	29
SpCas9-VQR	NGA	164	230	7,140	19	7
SpCas9-VRER	NGCG	6	4	3,852	0	
SpCas9-WT	NGG	633	2,027	46,571	73	14
SpRY	NRN	378	5,582	10,169	24	22
SpaCas9	NNGYRA	3	11	6,290	1	1
St1Cas9	NNAGAAW	27	15	4,716	11	1
Un1Cas12f1	TTTR	16	110	6,724	0	
spaCas9	NNGYRA	3	12	6,381	1	1
xCas	NGN	7	2,904	8,862	0	

### 2.5 PAM specificity impacts targetability

**FIGURE 4:**
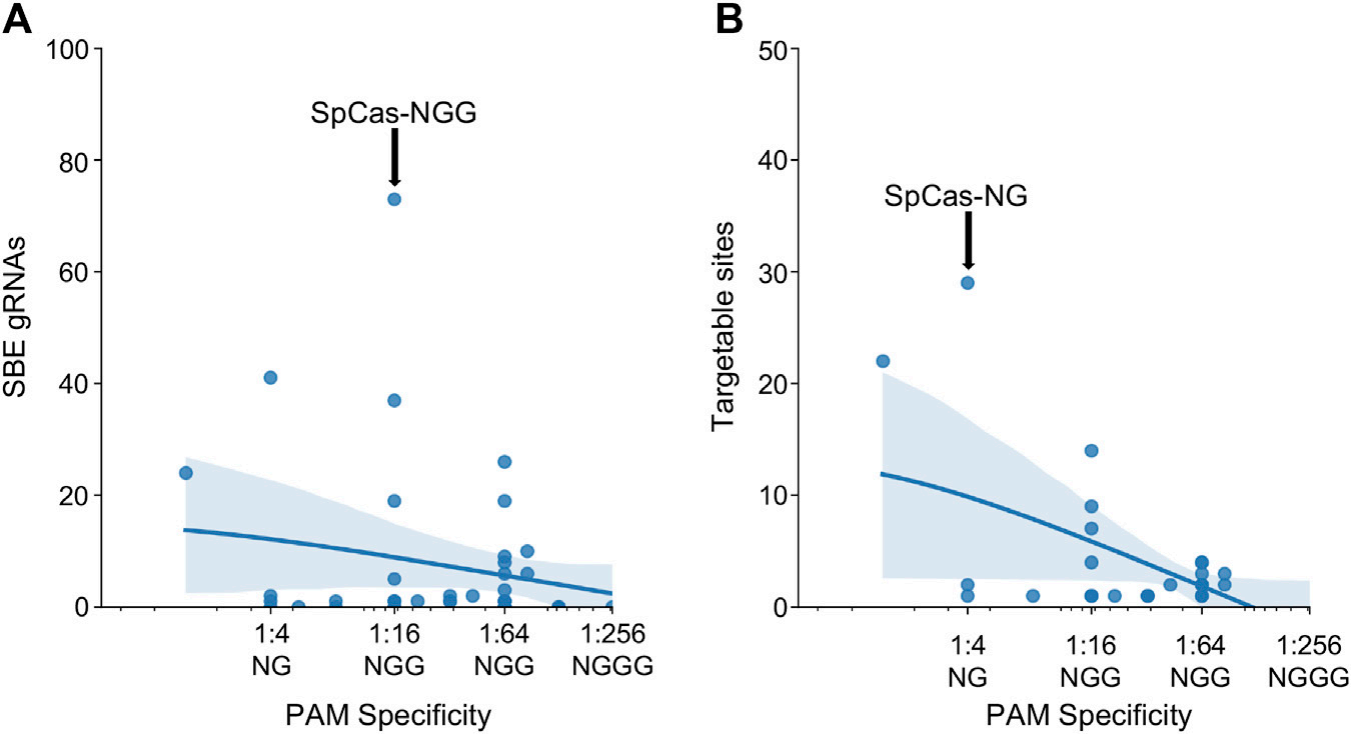
Increase PAM specificity leads to a decrease in SBE gRNAs and loss of targetable sites. The number of **(A)** SBE gRNAs and **(B)** targetable sites was plotted against the PAM specificity. The X-ticks are representative PAMs for each level of specificity. The blue line represents a y ∼ log(x) regression and the shadow indicates a 95% CI. A regression was performed for SBE ∼ log_10_(PAM-promiscuity) and found no significant correlation. Alternatively, Unique-Sites ∼ log_10_(PAM-promiscuity) found a significant correlation (*p* = 0.0039) with a modest *R* ^2^ = 0.26.

After investigating the effect of specific PAM mutations for SpCas9, SaCas9, and LbCas12a, the exploration was broadened to all PAMs for all Cas molecules found across the study (N = 43). The PAM tolerance of each enzyme was plotted against the number of SBE gRNAs (Figure 4A) and unique targetable sites (Figure 4B). The analysis shows that more specific PAMs, those with lower levels of degeneracy, have less SBE gRNAs and target fewer unique sites in HIV-1. A regression was performed for SBE ∼ log_10_(PAM-promiscuity) and found no significant correlation. Alternatively, Unique-Sites ∼ log_10_(PAM-promiscuity) found a significant correlation (*p* = 0.0039) with a modest *R* ^2^ = 0.26.

### 2.6 Numerical stability of estimates

**FIGURE 5:**
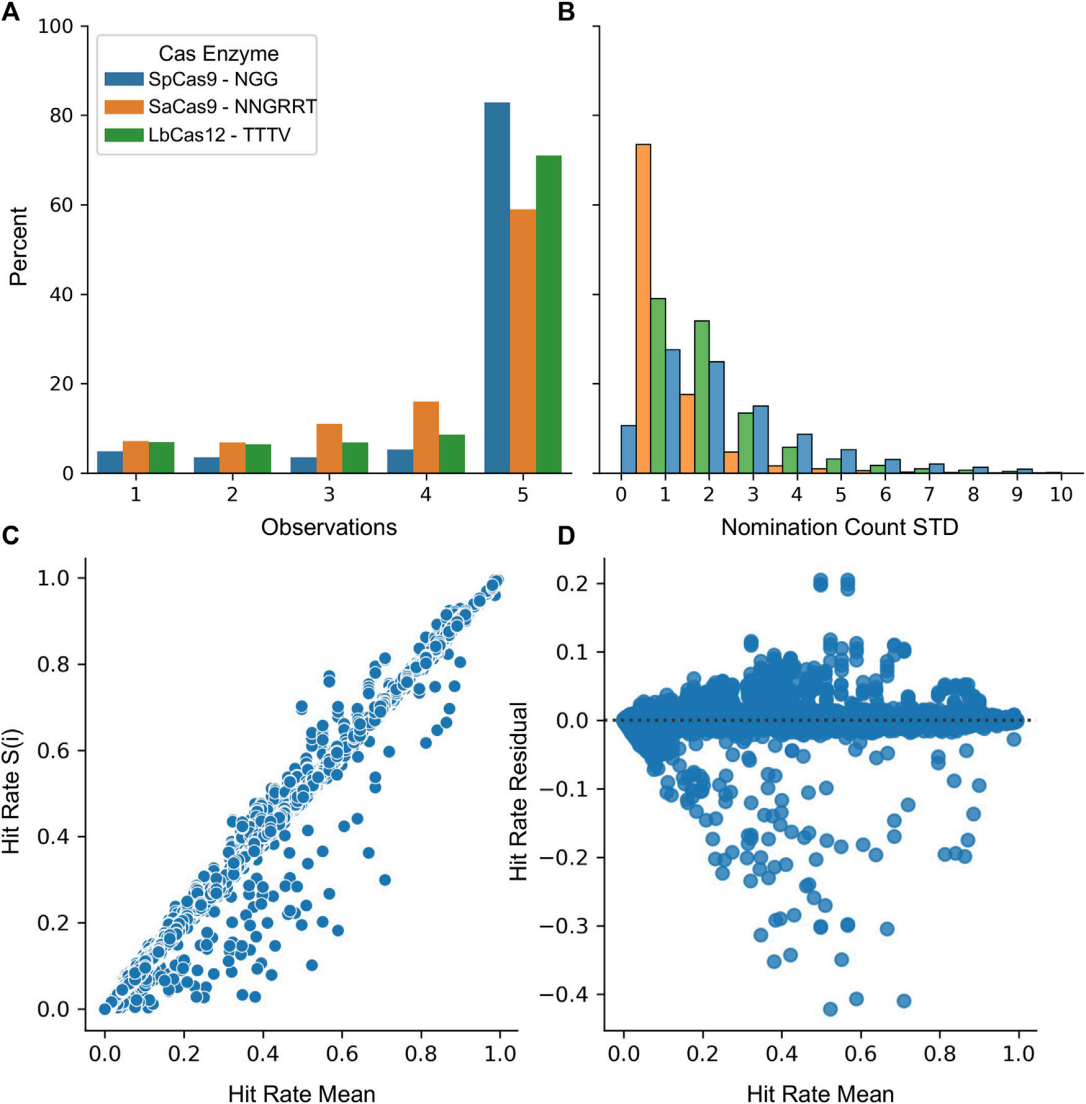
Nomination and validation stages are unimpacted by random samplings. **(A)** The number of samplings each protospacer was nominated in during cross-validation. **(B)** A histogram of the standard deviation across each sampling for each protospacer. For **(A)** and **(B)** SpCas9 is in blue, SaCas9 is in orange, and Cpf1 is in green. **(C)** The mean validation hit rate was calculated across all samplings (x) and then was plotted against the hit rate obtained in each S(i) sampling (y). **(D)** A residual plot for the regression of *Hit Rate S(i)* ∼ *Hit Rate Mean*.

Finally, the stability of the protospacers was evaluated for the SpCas9, SaCas9, and LbCas12a enzymes. A five-fold resampling was performed such that five different globally representative validation datasets were created along with the remaining nomination sets. For each of these samplings, the top 20,000 most common protospacers were collected. Figure 5A shows the likelihood of being nominated by 1, 2, 3, 4, or 5 samplings. Most protospacers (82% SpCas9, 58% SaCas9, and 71% LbCas12a) are nominated in all five samplings with only a minority (4% SpCas9, 7% SaCas9, 7% LbCas12a) being nominated in a single sampling. Figure 5B shows the variability of the count between samplings. The average standard deviation across the same protospacer was (3.0 SpCas9, 0.8 SaCas9, and 1.7 LbCas12a). Together, these results indicate that one is unlikely to miss high-count protospacers during the nomination phase due to the sampling seed.

Evaluation of the validation stage was performed by first nominating the top 40,000 protospacers from the entire LANL dataset. Then, this set was then evaluated against each of the five samplings. Figure 5C shows the correlation between the mean score and the score generated from each sampling. The mean absolute error was calculated as 0.000425 indicating the value from each sampling is incredibly close to the average value. Figure 5D shows the residual between each sampling and the mean assuming a linear relationship. Some structure is observable with an increase in model variability near the 50% rate. As the purpose of this technique is to draw from the upper 75%, this is less concerning.

## 3 Discussion

As the HIV-1 gene editing field advances, it is important to look for advances in other areas of gene editing and how they can be optimally adapted. The wider gene editing field is extending the capabilities of Cas molecules and this manuscript presents a framework under which to evaluate different trade-offs being introduced into Cas systems. This study focused on exploring three major questions: 1) how Cas editors impact the targetable HIV-1 genome, 2) how mismatch tolerance impacts broad spectrum potential vs. off-target risk, and 3) how novel PAM variants enhance targeting potential.

This evaluation finds that the wider PAMs variants are a boon to HIV-1 gene editing. The NNNRRT PAM variants of SaCas9-KKH increases the number of SBE gRNAs and increases the number of targetable HIV-1 sites. The Cas12 system is new and has had less published molecular engineering, we explored other natural Type V PAMs and found the broader PAMs have more SBE gRNAs and more unique sites. New variants of the LbCas12a protein will likely widen the PAM. For SpCas9, the story is more complex, SpCas-NG and SpRY decrease the number of SBE gRNAs but increase the number of valid HIV-1 targets.

Mismatch tolerance is also an important tunable parameter. Our analysis indicates that for all editors, the peak of usefulness is at a 2-mismatch tolerance. Tuning the system to be more specific by decreasing the tolerance limits the ability of the system to cope with HIV-1 genetic variability. Similarly, adjusting the system to permit more mismatches may lead to unwanted off-target impacts.

This analysis is limited in several ways and should not be considered an exhaustive search for optimal anti-HIV-1 gRNAs for any Cas enzyme. This pipeline does not consider important parameters like position-specific mismatch penalties, differences in on-target gRNA activity, and considerations like CG content, poly-N-mers, etc. It is also reliant on a relatively small public dataset. Instead, it is intended as a framework to evaluate the effect of choices like PAM motif and mismatch tolerance on the feasibly targetable regions of the HIV-1 genome. It points to broader PAMs and a modest promiscuity of two mismatches.

There are also considerations beyond targeting potential when choosing a Cas editor. The size is a particularly notable one as it influences packaging efficiency across many delivery systems (Liu et al., 2017a). In those cases, smaller editors like SaCas9 and LbCas12a are attractive options. Another consideration is the type of edit and location relative to the PAM. Type II editors tend to cut near the PAM site, often leading to the erasure of the PAM site during DNA repair making retargeting difficult and leading to resistance profiles (Wang et al., 2016c; De Silva Feelixge et al., 2018). Conversely, Type V editors cut on the opposite side to the PAM, lowering this likelihood.

With these parameters taken together, it is difficult to arrive at a single definitive answer for the best Cas editor for HIV-1 targeting. The broad choice will be influenced by experimental setup, delivery strategy, and outcome variables. This manuscript indicates that it is useful to integrate editors with novel Cas mutations into next-generation strategies. This manuscript also indicates that techniques to decrease the mismatch penalty below 2 will reduce broad applicability and attempts to increase it above 2 will increase off-target effects. It is still early in the field of Cas9 gene editing and there will likely be further developments and improvements to Cas editors. HIV-1 researchers should stay abreast of upcoming technology to lead in the field.

## 4 Materials and methods

### 4.1 Dataset

**FIGURE 6:**
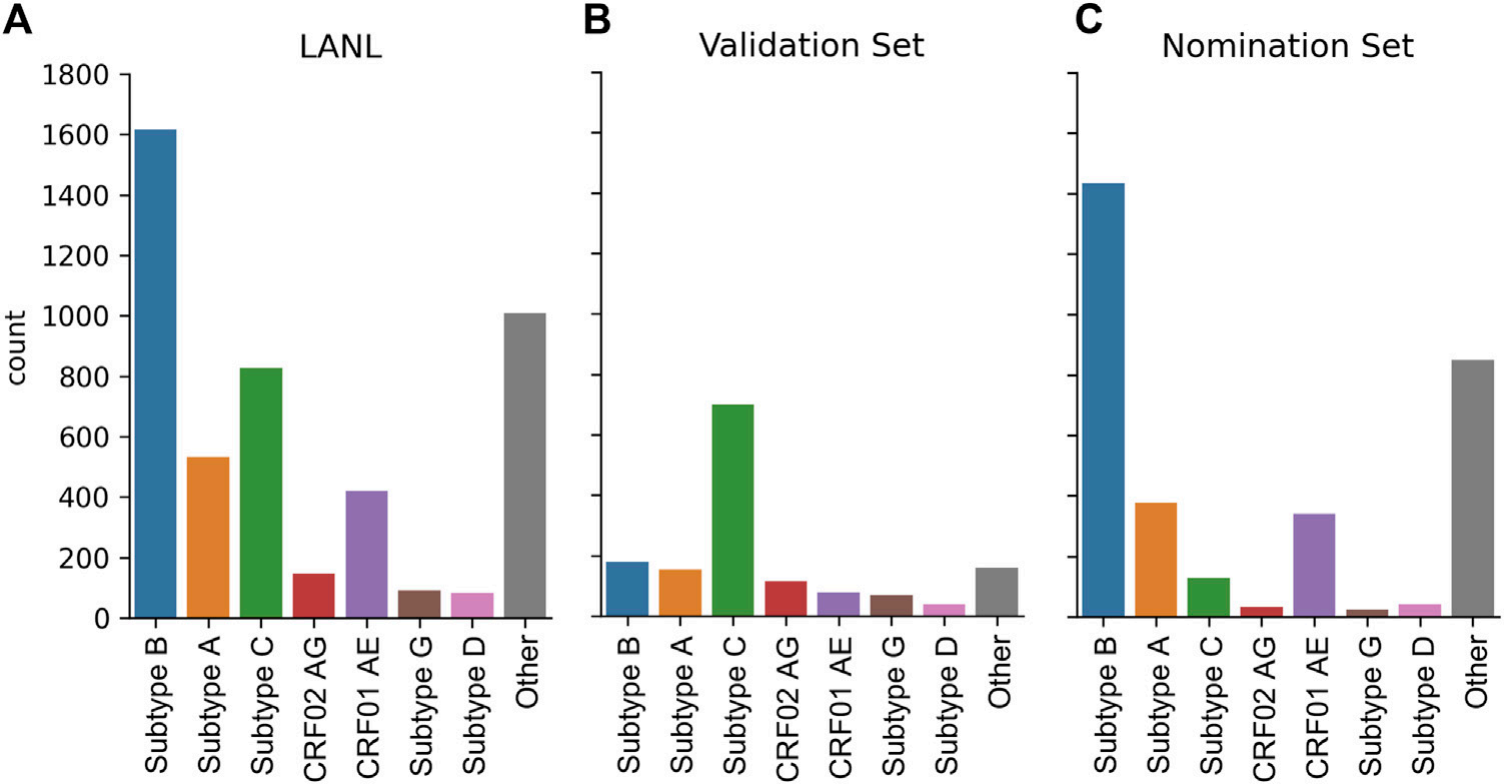
HIV-1 subtype distribution of full genomic sequences in the LANL dataset. **(A)** The entire 2021 LANL full genome dataset. **(B)** A subset of the LANL database randomly selected to be a globally representative validation dataset. **(C)** The remaining sequences used as a nomination set.

The dataset was constructed from the LANL full HIV-1 genome database from the Filtered web subset of full genome sequences including all sequences up to 2021 (last accessed 5/15/2023) and contains 4,725 sequences. However, this sequencing dataset is heavily biased towards Subtype B sequences (Figure 6A). To create a globally representative testing dataset, 1,497 sequences were randomly subsampled to match the proportions of global infections (Hemelaar et al., 2019) A: 10.3%, B: 12.1%, C: 46.6%, D: 2.7%, G: 4.6%, CRF01_AE: 5.3%, CRF02_AG: 7.7% (Figure 6B) and the remaining 3,228 sequences were used as the nomination dataset (Figure 6C).

### 4.2 Estimating target effectiveness

**FIGURE 7:**
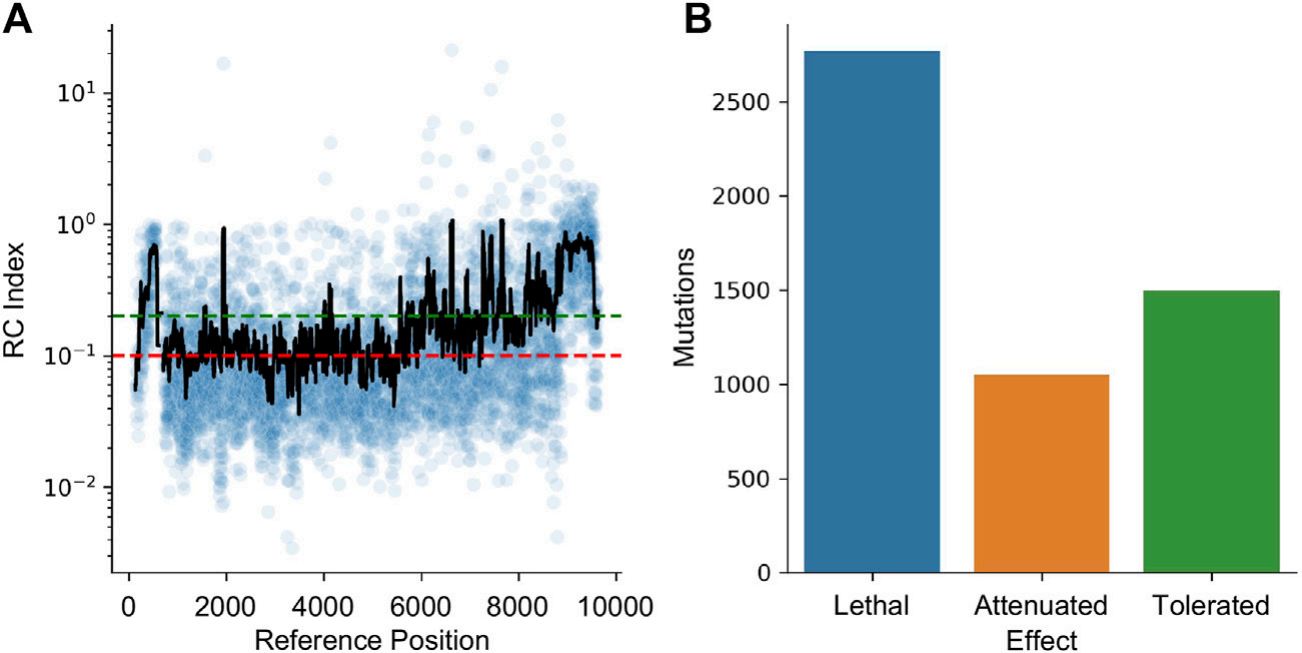
The effect of mutations on HIV-1 replication capacity. **(A)** The RC index of each induced mutation was plotted in blue. The black line represents a 40 bp rolling average centered on each point. The red line indicates the maximum RC index of a lethal mutation (0.1) while the green line indicates the minimum value of a tolerated mutation (0.2). Between the green and red lines indicates attenuated. **(B)** The number of lethal, attenuated, and tolerated mutations is plotted.

HIV-1 is a highly mutable virus and as such, the impact of edits may be muted by a lack of functional impact. To quantify this effect, a dataset of random mutations was used to quantify the impact of variability across the genome. The dataset from Al-Mawsawi et al. was obtained from the Retrovirology Additional File #6 (last access 5/15/2023) (Al-Mawsawi et al., 2014). This experiment used random mutagenesis and subsequent selection to determine the Replicative Capacity (RC) index of each mutation. In their experiments, the RC index was calculated as the ratio of the frequency of the mutant in the 2^nd^ passage to the frequency in the original infecting library; larger numbers indicating positive selection and smaller numbers indicating negative selection. The dataset was converted to HXB2 numbering and the RC index was averaged for all mutations reported within a 40 bpwindow centered at each position (Figure 7A). Using the cutoff from Al-Mawsawi et al., RC index below 0.1 is considered a lethal mutation, between 0.1 and 0.2 as attenuated, and above 0.2 as tolerated (Figure 7B). This cutoff found 2,771 sites where mutations are likely lethal, 1,052 sites that may lead to an attenuated phenotype, and 1,498 sites that are tolerant to mutations.

### 4.3 Nominate, diversify, narrow, and filter (NDNF) pipeline

#### 4.3.1 Nominate

The first step is to nominate potential protospacers from a collection of sequences. A custom python function was written to convert protospacer and PAM motifs into regular expressions targeting each strand. For example, the NGG SpCas9 PAM was converted into the regular expressions (.)[ACGT]GG and CC[ACGT](.). These regular expressions were then used to extract all extant protospacers in the nomination dataset. This was done independently for each Cas PAM motif. In our testing of 3,228 sequences, this yielded 5,000–3,00,000 unique protospacers depending on the promiscuity of the PAM.

#### 4.3.2 Diversify

As the testing and nomination datasets are distinct, and the potential variability space of HIV-1 is vast, it is important to explore potential protospacers beyond those in the nomination dataset. The diversify step addresses this by randomly mutating the set of protospacers to create new ones. Custom python scripts (provided at https://github.com/DamLabResources/Cas_HIV_hits) were used to randomly change protospacers, keeping only unique ones. This randomization was run until there were 40,000 unique protospacers. This number of protospacers was chosen as a balance between an exhaustive search of all possible protospacers and the observation that rare protospacers are unlikely to be broadly targeting.

#### 4.3.3 Narrow

The 40,000 unique protospacers were then evaluated against the validation dataset. CasOffinder was used to find all potential binding sites with mismatches (MM) in the protospacer; where MM was set to 2 for all experiments unless otherwise stated. All mismatch searches were performed with CasOffinder v2.4.1 (Bae et al., 2014). Due to limitations in CasOffinder v2.4.1 RNA and DNA bulges were not considered. Only protospacers which have matching sites within at least 75% of the validation dataset were considered *broad* and moved into the next stage. By introducing this filtering step, the computationally expensive search of the human genome can be limited to only those protospacers that are likely to be Safe, broad, and effective.

#### 4.3.4 Filter

The broad-spectrum protospacers were then evaluated for off-target likelihood. Again, CasOffinder was used to search for all potential binding sites with 2 or fewer mismatches unless otherwise stated. A protospacer was deemed *safe* if there were 0 matching sites in the human genome (GRCh38, last access 5/15/2023) and unsafe if it had 1 or more hits.

### 4.4 Stability

The stability of this method to different samplings was tested through 5-fold resampling. In this, 5 different globally representative samplings were generated. The nomination stability was measured by nominating 40,000 protospacers from each of the folds and comparing the likelihood of being nominated in any given sampling and the deviation in the number of counts. The validation hit rate was tested by first nominating 40,000 protospacers from the entire dataset, to generate a consistent set of protospacers to evaluate. Subsequently, the 40,000 protospacers were tested against each of the five samplings to measure the difference between the hit rate measured in any given sampling to the mean of all samplings.

## Data Availability

The datasets presented in this study can be found in online repositories. The names of the repository/repositories and accession number(s) can be found below: https://github.com/DamLabResources/Cas_HIV_hits.
